# Phytochemical profiling and cellular antioxidant efficacy of different rice varieties in colorectal adenocarcinoma cells exposed to oxidative stress

**DOI:** 10.1371/journal.pone.0269403

**Published:** 2022-06-03

**Authors:** Akanksha Tyagi, Umair Shabbir, Xiuqin Chen, Ramachandran Chelliah, Fazle Elahi, Hun Ju Ham, Deog-Hwan Oh

**Affiliations:** 1 Department of Food Science and Biotechnology, College of Agriculture and Life Sciences, Kangwon National University, Chuncheon, South Korea; 2 Department of Biological Environment, College of Agriculture and Life Sciences, Kangwon National University, Chuncheon, South Korea; Bangabandhu Sheikh Mujibur Rahman Agricultural University, BANGLADESH

## Abstract

In the present study, white (Baegilmi), brown (hyunmi) and black (chalheugmi) Korean local rice varieties ethanol extracts were analyzed for *in-vitro* antioxidant assays (ABTS, FRAP and DPPH), cellular antioxidant activities (CAAs) and phenolic phytochemicals content. The highest antioxidant assays, phenolic, flavonoid and anthocyanins content were identified among the free fractions of black rice. Phenolic phytochemicals were detected and quantified using the ultra-high-performance liquid tandem chromatography quadrupole flight mass spectrometry (UHPLC-Q-TOF-MS^2^). Which indicated the richness of several phytochemicals like ascorbic acid, vanillic acid, p-coumaric acid, catechin, epigallocatechin and quercetin in black rice than in other rice samples. The cellular antioxidant activities (CAA) of black rice were found equivalent to that of ascorbic acid, the standard employed in the assay. The CAAs of free fractions were as follows: white rice < brown rice < black rice. These findings are significant for enhancing human health through increased consumption of black and brown rice in the development of functional food products.

## 1. Introduction

Rice (*Oryza sativa*) is considered a staple food in various nations, notably those in Asia. Rice is also one of the most essential and reliable crops for human consumption as it can thrive in damp areas. In contrast to other cereals, rice has a tough outside husk that protects the kernel. The rest of the endosperm is referred to as polished rice and is typically consumed when bran and embryo are removed. Rice grain is a key source of carbohydrates, protein, and other essential nutrients. Pigmented rice is whole grain rice with an unbroken bran layer and varied coloured pericarps such as black, brown, and red. Consumer interest in coloured rice types has shifted due to their possible health advantages, which have been linked mainly to the presence of polyphenolic compounds. Simple phenolic acids to complex polyphenols like anthocyanins are among the phenolic chemicals found in coloured rice types. On the other hand, they include significant amounts of dietary fiber and plant phytochemicals like tocotrienols, tocopherols, oryzanols, vitamins, and phenolics that improve individual health and well-being [1]. Endosperm and bran/embryo regions of the complete rice grain include phytochemicals in free, soluble-conjugated, and bound forms.

Epidemiological research has indicated that rice antioxidants may be responsible for the reduced prevalence of chronic illnesses in rice-consuming countries [2]. Rice phenolic compounds such as ascorbic acid [3], quercetin [4], and p-coumaric acid [5] are well known in the literature for their strong antioxidant as well as oxidative stress-reducing effects in *in-vitro* and animal models. Additionally, several studies have described the beneficial effects of pigmented rice as an anti-inflammatory [6], increased mRNA expressions of fatty acid metabolism-related genes and decreased hyperglycemia and hyperlipidemia in rats [7]. One of the most abundant phytochemical groups in whole grains is phenolics, which act as radical scavengers to reduce the frequency of oxidative stress-induced damage to major biological components. Many studies have examined the phytochemical content and antioxidant activity of different rice types, including the presence of gamma-aminobutyric acid (GABA), phenolic acids, oryzanol, tocotrienol and flavonoids [8]. At the same time, several factors can affect the content of these nutrients and bioactive substances, including rice type, production environment, geographic origin, and postharvest treatments or processing conditions.

To analyze dietary antioxidants, various *in vitro* techniques are frequently employed nowadays; however, their overall effectiveness was questioned because biological systems cannot be predicted only by using *in vitro* chemical studies conducted under non-physiological circumstances [9]. It was previously discovered that a grain’s antioxidant activity was related to its total phenolic content. Because most phenolics in food have relatively poor bioaccessibility and bioavailability, it is critical to determine the antioxidant activity of these phenolics under physiological circumstances [10]. Thus, cell-based assays are gaining appeal as a more therapeutically relevant method that fits alongside *in vitro* testing, animal feeding research, and human clinical trials. The quantitative Cellular Antioxidant Activity (CAA) assay is one example of such a test [11]. CAA test is effective for evaluating the bioavailability of dietary antioxidants, as it may take into account many other factors, including cellular absorption and metabolism. Consumers who prefer to get antioxidants with a high CAA content from rice rather than large dosages of antioxidants from diet supplements would be very interested in the CAA of rice with varied hues.

Antioxidant treatment has been a helpful technique in understanding the complex genesis of chronic illnesses as well as developing new remedies to reduce the adverse effects of medication therapy [12, 13]. Rice-derived polyphenols and their antioxidant properties in global rice cultivars have been widely researched. However, research on the antioxidant potential of rice cultivars cultivated in south Korea is scarce. Investigating the phenolic content and antioxidant activity of coloured or non-coloured rice cultivars cultivated in Korean soil might show their potential as a functional food and help Korea enter the worldwide market.

As a result, the ultimate goal of our research was to give the knowledge to measure the quality of these phytochemical antioxidants in different rice types to suit the demands of rice growers and consumers. The specific aim of our study was (1) to reveal the antioxidant activity, phenolic, flavonoids and anthocyanin content of different rice varieties, (2) to demonstrate cellular antioxidant efficiency of rice and (3) detection of phytochemical compounds present in different local rice varieties using UHPLC-Q-TOF-MS^2^.

## 2. Experimental materials and methods

### 2.1. Research samples

The current study employed one variety of each type of rice (*Oryza sativa L*.), namely chalheugmi (black rice), Baegilmi (white rice), and hyunmi (brown rice), based on their availability, consumption and cultivation in the local area. All samples were purchased from the local market, Chuncheon Gangwon-do, South Korea (S1 Fig, S1 Table). After being crushed into a powder using an electric grinder, the samples were sieved via mesh size 40 to remove any remaining dust or debris. The samples were stored at -20 °C before the further procedure.

### 2.2. Chemicals and cultures

Ethanol, acetonitrile, acetone, methanol, phosphate-buffered saline (PBS), sodium carbonate, penicillin-streptomycin, sodium hydroxide, anhydrous sodium acetate, hydrochloric acid, trypsin EDTA, Williams’ Medium E, potassium persulfate, acetic acid, sulfuric acid, gentamicin, insulin, L-glutamine, Dulbecco’s modified eagle medium (DMEM) and advanced DMEM were obtained from Daejung chemicals and metals Co., Ltd, South Korea and Life Technologies (Grand Island, NY, USA). The Phenolic standards and other chemicals like ABTS (2,2′Azino-bis (3-ethylbenzothiazoline-6-sulfonic acid), 2′,7′-Dichlorofluorescin diacetate or 2′,7′-dichlorodihydrofluorescein diacetate (DCFH-DA, ≥ 97% purity), TPTZ (2,4,6-Tris(2-pyridyl)-s-triazine), caffeic acid, ferulic acid, 2,2 Diphenyl-1-picrylhydrazyl (DPPH), gallic acid, Folin–Ciocalteu reagent, quercetin dehydrate (≥ 98% purity), p-Coumaric acid, (+)-catechin hydrate, dimethyl sulfoxide (DMSO); 2,2′-azobis(2-amidinopropane) dihydrochloride (ABAP), and Trolox (6-hydroxy-2,5,7,8-tetramethylchroman-2-carboxylic acid) were purchased from Sigma, South Korea. Analytical grade chemicals and reagents were used.

### 2.3. Sample preparation

#### 2.3.1. Preparation of ethanolic extracts

Extraction was done using our previous procedure [14] with some modifications. Hexane was used to remove lipids from the samples. Ground materials were defatted three times with hexane (1:5, w/v, 2 h). Before usage, defatted flour was stored at -20 °C. After defatting samples (5 g), soluble phenolics were extracted in an orbital shaker (DAIHAN scientific RK-2D, Wonju, Korea) with 70% ethanol (1:20 w/v) for 1 h at 50 °C. This method was continued till the third extraction. The extracts were centrifuged at 4000 rpm for 10 min with a Union 32R plus centrifuge (Hanil Science Industrial, Incheon, Korea). The supernatants were freeze-dried after evaporation at 50 °C to remove any remaining liquids. Solids were lyophilized and then kept at -20 °C before reconstituting with ethanol. The sample stock solution was produced at a 1 mg/mL concentration throughout the study.

### 2.4. Measurement of Total Anthocyanin Content (TAC)

After some modifications, the anthocyanins were determined using a previous methodology [15]. In brief, 0.1 g of each freeze-dried sample was dissolved in 10 mL of 60% ethanol containing 1% citric acid, completely mixed with a vortex, and absorbance at 535 nm was measured using a spectrophotometer (Evolution 201, Thermo, Waltham, MA, USA). TAC was calculated as mg C3G Equiv./100 g, DW using cyanidine 3-O-glucoside chloride (C3G) as a reference.

### 2.5. Measurement of Total Phenolic Content (TPC)

The measurement of TPC was done with the Folin-Ciocalteu (FC) colourimetric method using the previously reported method [14] after some modifications. In brief, the FC reagent was mixed with the sample extract or standard ferulic acid solution (100 μL) for a brief period of 6 min. Then in the next step alkalizing the mixture using 1 mL of Na_2_CO_3_ 700 mM solution. A SpectraMax i3 plate reader was used to measure the absorbance after 90 min in the dark (Molecular Devices Korea, LLC). As a result of the standard gallic acid curve, the TPC of the sample was calculated and represented as mg GAE Equiv./100 g, DW (mg of gallic acid equivalent per 100 g of the sample).

### 2.6. Measurement of Total Flavonoid Content (TFC)

The 24-well microplate technique was used to quantify the TFC of ethanol extracts as defined by the previously reported method [14] with minor changes. Shortly, extracts were mixed with distilled water and NaNO_2_ (50 g/L) in quantities of 200 μL each. Incubation for 5 min was followed by adding AlCl_3_ (75 μL; 100 g/L) to the mixture. Later, after 6 min, 600 μL of distilled water and 500 μL of 1 M NaOH were added. Following 6 min, 600 μL of distilled water and 500 μL of 1 M NaOH were added. The absorbance at 510 nm was measured using a SpectraMax i3 plate reader (Molecular Devices Korea, LLC). Accordingly, findings were reported in mg catechin Equiv./100 g, DW (mg catechin equivalent per 100 g of sample).

### 2.7. Antioxidant activity assays

#### 2.7.1. DPPH radical scavenging activity

DPPH assay was examined by the procedure mentioned previously [14] with slight alterations. In conclusion, 100 μL of the sample extract, standard (Trolox), or blank was combined with freshly prepared 100 μL of 500 μM DPPH solution (dissolved in methanol) using a 24-well microplate and maintained at RT for around 30–40 min in triplicates. Quantification was done at 515 nm absorbance, and Trolox was used for the baseline curve. Values of DPPH were demonstrated as IC_50_ (μg/mL).

#### 2.7.2. ABTS radical scavenging activity

ABTS activity was measured using our earlier protocol [14]. Values of ABTS were demonstrated as IC_50_ (μg/mL).

#### 2.7.3. Ferric Reducing Antioxidant Power (FRAP)

FRAP was examined following the documentation in previous reports [14] with some improvements. In short, extracts were mixed with the FRAP solution, which was produced using 0.3 M acetate buffer (50 mL, pH 3.6), 5 mL of Tripyridyl Triazine (TPTZ) solution (10 mM of TPTZ in 40 mM of HCl) and 5 mL of FeCl_3_·6H_2_O (20 mM). The mixtures were incubated at 37 °C for 10 min, and the values were quantified at 593 nm absorbance. All of these results were expressed as IC_50_ (μg/mL).

### 2.8. Cell-based antioxidant activity assay

#### 2.8.1. Cellular Antioxidant Activity (CAA) assay

Oxidation-sensitive DCFH-DA probes were used to measure reactive oxygen species (ROS) within cells by previous researchers [16], so after some modifications, we have conducted the CAA assay. Briefly, Caco-2 cells were cultured overnight in black 96-well microplates at a density of 6 ☓ 10^4^ cells per well in a 5% CO_2_ Incubator at 37 °C. After overnight growth, 2 h of pretreatment with varied doses of sample extracts (0.5–5 mg/mL) and 100 μL of DCFH-DA (25 mol/L) was provided in each cell. Later, 50 μL of PBS was used to wash the cells. As a final step, inoculation of each well, except for the blank well, was carried out with the addition of 100 μL DMEM (freshly prepared 600 μmol/L of ABAP radical) to each well.

The decrease in fluorescence was calculated to determine the effectiveness of antioxidants in cell lines. Each treatment received 12–13 fluorescence response measurements during the 1 h analysis, resulting in curves. The findings for CAA were analyzed using the standard quercetin and reported as quercetin (QE) Equiv./100 g as well as in percentage and represented as CAA%. The following formula was used to compute the CAA values of phytochemicals at each concentration.


$$\text{CAA}\;\text{unit}=1-\frac{\text{integrated}\;\text{area}\;\text{of}\;\text{the}\;\text{fluorescence}\;\text{versus}\;\text{time}\;\text{curve}\;\text{sample}}{\text{integrated}\;\text{area}\;\text{of}\;\text{the}\;\text{control}\;\text{curve}}$$

#### 2.8.2. Cell viability assay

The EZ-cytox test at our laboratory was used to assess the cytotoxicity of the sample extract. WST chemical is exclusively active in live cells, found in the mitochondrial respiratory chain. Caco-2 cells were plated in growth media at a density of 4 ☓ 10^4^ cells per well in 96-well microplates. Cells were washed with PBS after 24 h of maintenance at 37 °C with 5% CO_2_. Then, 100 μL of growing media containing varying amounts of sample extract (0.5–20 mg/mL) was added. Those in the control group received a medium that did not contain sample extract as a supplement. Each sample was maintained at 37 °C for 24 h with 5% CO_2_ before adding 10 μL of WST-1 solution. The absorbance was observed at 455 nm at 37 °C. It is cytotoxic if a sample extract concentration reduces cell viability by more than 10%.


$$\%\;\text{of}\;\text{cell}\;\text{viability}=\frac{\left(\text{mean}\;\text{absorbance}\;\text{in}\;\text{test}\;\text{well}\right)}{\left(\text{mean}\;\text{absorbance}\;\text{in}\;\text{control}\;\text{well}\right)}\times 100$$

### 2.9. Phytochemicals identification using UHPLC-Q-TOF-MS^2^

Phytochemicals of rice varieties were identified using UHPLC-Q-TOF-MS^2^. Stock samples (1 mg/mL) were used for detection, just like in previous experiments. Millex syringe filters (Merck KGaA Darmstadt, Germany) with 0.25-micron pore size were used to filter the samples and supernatants (1 mL) were separated and transported to MS vials for further analysis. Positive (ESI+) and negative (ESI−) modes of mass spectrometric analysis were used as described earlier in our previous study [14].

### 2.10. Statistical analysis

A minimum of three analyses were carried out for all the experiments conducted in the present research. GraphPad Prisma 8.0 was used to analyze the obtained data. The one-way variance analysis (ANOVA) and the Tukey’s test at the significant level of at least 0.05 were considered statistically significant. Average Standard Deviation (SD) was used to explain the findings.

Pubchem, ChemSpider and XCMS online (Metlin) were used to identify compounds empirically. (https://pubchem.ncbi.nlm.nih.gov/), (http://www.chemspider.com/) and (https://xcmsonline.scripps.edu). Multivariate statistical studies and heat maps were carried out using the ClustVis software (http://biit.cs.ut.ee/clustvis/). Principal component analysis (PCA) was done using Origin 2021 software. To compare the changes among rice samples, the PCA method was employed. ClustVis and Origin were used to create Heat maps & PCA utilizing peak regions of samples. Past 4.0 software was used to compare the correlations among antioxidants and phenolics.

## 3. Results and discussion

### 3.1. TPC, TFC and TAC of ethanol extracts

Phenolics are a kind of phytochemical that contains one or more hydroxyl groups from aromatic rings, and their concentration has been connected to grain antioxidant properties [17]. Total phenolics, flavonoid, and anthocyanin content of all three tested local Korean rice varieties were presented in Table 1. When the total free phenolic content of rice types was evaluated, it was seen that pigmented rice varieties had much greater TPC than non-pigmented rice varieties. The TPC among rice samples varied from 64.80 ± 0.87 to 503.14 ± 1.07 mg GAE/100 g, DW. TPC levels were found highest in black rice (503.14 ± 1.02 mg GAE Equiv./100 g), followed by brown rice (150.48 ± 0.95 mg GAE Equiv./100 g), and least content in white rice (64.80 ± 0.87 mg GAE Equiv./100 g). As shown in a recent study, free phenolics have been significant contributors to total TPCs in rice [10]. In our research, TPC was higher than reported in the previous research [18]. Phenolics of black and brown rice were known for a wide variety of beneficial effects, including antioxidant effect, type 2 diabetes, anti-inflammatory effect, inhibiting α -glucosidase and α-amylase activity, prevention of heart and cardiovascular diseases, protection of endothelial cells and in reducing the risk of cancer, neurodegenerative diseases, hypertension, and obesity [19].

**Table 1:** Table representing TPC, TFC and TAC of different rice varieties.

Sample	TPC(mg Gallic acid Equiv./100 g, DW)	TFC(mg catechin Equiv./100 g, DW)	TAC (mg CG3 Equiv./100 g, DW)
**White rice**	64.80 ± 0.87^c^	73.44 ± 1.87^c^	ND
**Brown rice**	150.48 ± 0.95^b^	136.63 ± 1.0^b^	13.75 ± 1.21^b^
**Black rice**	503.14 ± 1.02^a^	479.95 ± 0.78^a^	109.81 ± 1.37^a^

The findings are presented as the mean ± SD of triplicate. Statistically, significant differences are represented by different alphabetical letters in each column (Tukey and Duncan test p ≤ 0.05). DW stands for dry weight sample.

Flavonoids are a category of phenolic compounds with high antioxidant action and are related to a reduced risk of chronic illnesses. TFC of rice-free fractions varied from 73.44 ± 1.87 to 479.95 ± 0.78 mg catechin Equiv./100g, DW. Black rice consists of the highest TFC, identical to the findings in free TPC content (Table 1). According to the study, flavonoids were the main phenolic class in rice, notably in free black and brown rice fractions; this corresponded to the results provided in a previous study [20]. Our results indicate that free phenolic and flavonoid content in rice were higher in black & brown rice (pigmented) as compared to white or non-pigmented rice.

As we know, flavonoids and phenolics are covalently linked to cell wall structures through ester linkages, which cannot be digested immediately and may endure stomach processing and make it to the colon undamaged. They are broken down in the colon by bacteria, which may release the bound phenolics to perform favorable biological actions locally. So bound and free phenolics and flavonoids are all absorbed by the body and exert their beneficial effects. Moreover, our findings are consistent with earlier research, in which cereals showed higher free phenolics and flavonoid contents. In our research, we observed greater total TFC compared to previous studies [10, 21]. These variations in rice values amongst researchers might be attributed to changes in the growing landscape, genotype, and meteorological circumstances. In addition, various extraction solvents and methods can substantially impact the phenolic content of cereals.

Anthocyanins have been shown to have a strong antioxidant capability. Anthocyanins were major hydrophilic flavonoids detected in cereal grains. In our research, black rice showed the highest anthocyanin content (109.81 ± 1.37 mg CG3 Equiv./100 g, DW), followed by brown rice (13.75 ± 1.21 mg CG3 Equiv./100 g, DW). In contrast, no anthocyanin content was detected in white rice, which shows that coloured rice is rich in anthocyanin content with more vital antioxidant activities (Table 1). Furthermore, the current studies revealed that the anthocyanin concentration in rice was connected to the colour of the grain. Anthocyanins are thought to be the major differentiating factor between coloured and white rice. Anthocyanin intake has been linked to several health advantages, including neuroprotection, glycemic management, anticancer, anti-hypertension, and immunological response enhancement. Our research found higher anthocyanin content in coloured rice than previously reported by Rao et al. [22].

### 3.2. *In-vitro* antioxidant analysis (DPPH, ABTS, & FRAP)

**Fig 1:**
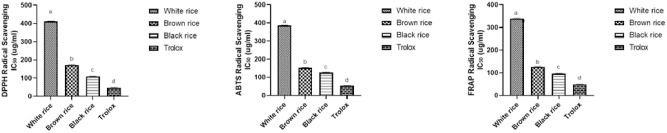
Ethanol extracts from rice cultivars have antioxidant properties. DPPH radical scavenging IC_50_ values, ABTS radical scavenging IC_50_ values, and FRAP radical scavenging IC_50_ values. A minimum of three analyses were carried out. Significant differences are indicated by different lower case letters (p < 0.05).

Due to their antioxidant action, phenolic compounds are usually regarded as desirable components for human health. For this study, we chose to employ the DPPH, ABTS and FRAP tests since they examine distinct mechanisms of antioxidants, with the former being based on both hydrogen & electron transfer reactions, while the latter being based only on the electron transfer reaction. Rice extracts are potent antioxidants because of a variety of processes, including metal ion chelation, reducing capacity, free radical scavenging, and prevention from lipid peroxidation [23]. Fig 1 shows the IC_50_ values of ABTS, FRAP and DPPH for all three local Korean rice varieties ethanol extracts.

It was shown that the colour of the rice had a significant effect on DPPH activity (p < 0.05). The lowest IC_50_ values of DPPH activity were observed in black rice extract (109.617 ± 0.74 μg/mL), followed by brown (172.391 ± 0.56 μg/mL), and the highest in white rice (412.677 ± 0.85 μg/mL) extracts, respectively, in comparison to the common antioxidant compound, Trolox (47.095 ± 2.74 μg/mL). Similar to DPPH, ABTS showed comparable trends in analyses. Black rice had the lowest IC_50_ (127.741 ± 0.25 μg/mL) of ABTS activity, followed by brown and white rice (152.294 ± 1.74 and 386.716 ± 0.95 μg/mL) in comparison to the common antioxidant compound, Trolox (53.602 ± 1.04 μg/mL). FRAP was also found highest in black rice with the lowest IC_50_ values, 97.591 ± 1.36 μg/mL, followed by brown rice 126.112 ± 0.82 μg/mL and white rice extract (340.082 ± 1.54 μg/mL), respectively, in comparison to the common antioxidant compound, Trolox (49.032 ± 1.97 μg/mL). This study’s antioxidant activity results demonstrate that there are considerable variations between pigmented and non-pigmented rice cultivars. Based on the findings of this study, we may deduce that white and brown coloured rice varieties had a poorer free radical scavenging ability than black rice. Our findings are consistent with our TPC, TFC, and TAC levels, with black rice having the highest total phenolic, flavonoid, and anthocyanin content, followed by brown and white rice.

Previous research has shown that the content of total phenolics, flavonoids and anthocyanins in rice grains is favourably associated with antioxidant activities [24]. Our findings were also found higher than a few earlier reports [19, 25]. This suggests that black rice is a better source of antioxidants than brown and white rice, which is more commonly consumed in our diet. More rice types have been produced as nutritious meals in recent years, and they are becoming increasingly popular among consumers. However, the reduced antioxidant activity of white rice compared to coloured rice may be due to the loss of the outer bran layer during milling, which was discovered to have higher quantities of phenolic compounds with antioxidant properties.

### 3.3. *Ex-vivo* cell viability assay and CAAs

#### 3.3.1. Cell viability assay

Food extracts must be tested for their cytotoxicity before they are consumed in any way. In Caco-2 cells, the cytotoxic impact of rice extracts at doses ranging from 0.5 to 20 mg/mL was studied using the Ez cytox assay kit; a 12 h incubation of the extract yielded a cell viability result as shown in S2 Fig. After raising the concentration even up to 20 mg/mL, it was found that cell viability did not significantly decrease. Using 0.5–20 mg/mL doses, no significant changes were detected in cytotoxicity assays. A 12 h experiment showed that 98–99% of the cells in the extract were viable, which was similar to the control, indicating that the extracts were not cytotoxic. It was observed that our results were comparable to those provided earlier [26].

#### 3.3.2. *Ex-vivo* CAAs assay

**Fig 2:**
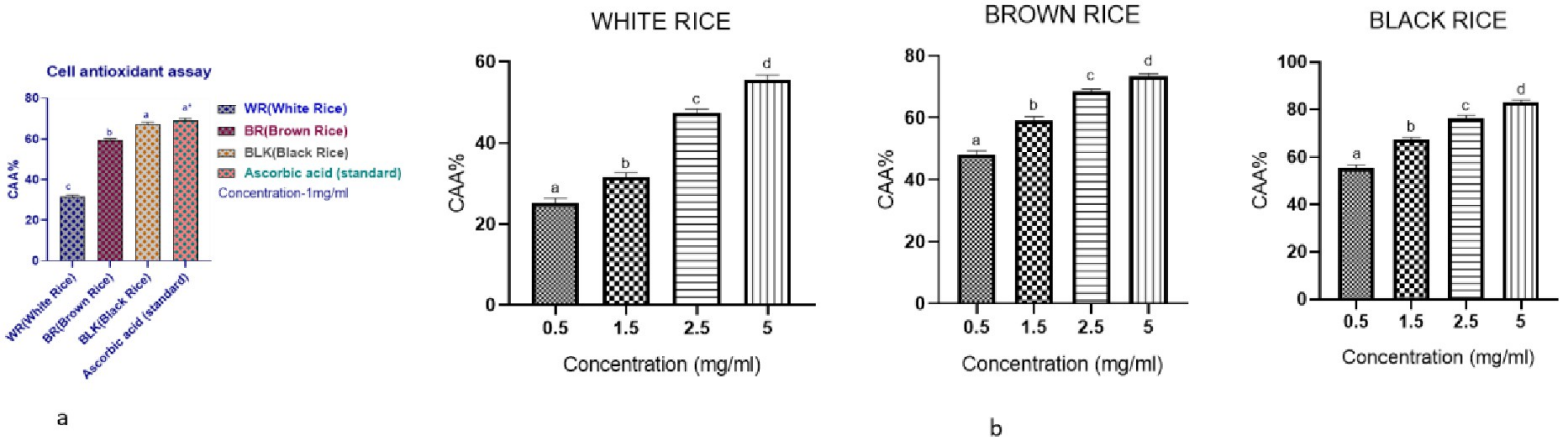
(A) CAA graph of rice extracts at 1 mg/mL. (B) Rice extracts at 0.5–5 mg/mL inhibited peroxyl radical-induced DCFH oxidation to DCF and ROS production in Caco-2 cells. Using one-way ANOVA, the data were reported as means ± standard deviations (n = 3). Tukey’s test at p < 0.05 shows significant differences between the columns using different alphabets (a–d). Note: (DCFH: 2’,7’–dichlorofluorescein diacetate, DCF: 2’, 7’–dichlorofluorescein, ROS: Reactive oxygen species, CAA- Cell antioxidant assay).

The CAA analysis was used to assess rice cellular antioxidant capability. Traditional chemical antioxidant tests are less physiologically relevant than CAA assays. Animal or human research is the most effective antioxidant approach; they are, however, costly and time-consuming. In contrast, the CAA assay is a low-cost and reasonably quick approach for evaluating foods and dietary supplements for antioxidants. Additionally, Caco-2 cells are also known to have similar morphology markers enzymes, tight junction, microvillar structure, and permeability as small intestine epithelial cells. So they are a good model nowadays for modeling the intestinal barriers in cell assays. In our analysis, Caco-2 cells were treated with different rice extracts to see if they had any effect on intracellular ROS. A DCFH-DA fluorescent probe is employed in our research as an indication of ROS for oxidative stress; intracellular esterases degrade DCFH-DA to 2′,7′-dichlorofluorescein following passive diffusion into cells, which is not fluorescent (DCFH). DCFH, confined inside cells, is oxidized by ROS and becomes fluorescent 2′,7′-dichlorofluorescein, a fluorescent dye (DCF) [27]. Oxidative stress arises when the cellular antioxidant defense mechanism cannot account for ROS generation. A bioactive substance can be used to delay this process, preventing DCF from being produced. Our rice extracts were tested in Caco-2 cells for their ability to combat oxidative damage. Our research used an intracellular oxygenating compound, ABAP, to mimic oxidative stress in cells. ABAP at a concentration of 600 μmol/L was found to be the optimum concentration for oxidation. Fig 2 depicts the oxidation kinetics of DCFH caused by peroxyl radicals. CAA values in rice extracts at 1 mg/mL were highest in black rice (98.93 mg QE Equiv./100 g or 67.34%), followed by brown (62.58 mg QE Equiv./100 g or 59.12%) and white rice (29.39 mg QE Equiv./100 g or 31.67%) rice respectively, as shown in Fig 2A. Later, a dose-dependent ROS reduction was also observed as shown in Fig 2B; CAA values rose from 25.42 ± 1.25% to 83.16 ± 1.53% with increasing concentrations from 0.5 mg/mL to 5 mg/mL. It has been demonstrated that rice phenolic extracts have high antioxidant activity. As the concentration of our rice extracts increased, the degree of inhibition followed a curvilinear pattern. A comparable effect was previously discovered in a few studies [11, 28]. In this research, the findings of the CAA indicated the high cell antioxidant activity or high inhibition of ROS by pigmented rice, especially black rice, compared to white rice. This demonstrates that the black rice variety used in this study can be a useful material for functional foods to avoid excessive reactive oxygen species-induced chronic diseases.

### 3.4. UHPLC-Q-TOF-MS^2^ detection

When it comes to accurately identifying and measuring a wide variety of components, UHPLC-Q-TOF-MS^2^ is regarded as the gold standard. The phytochemicals in rice samples were identified using this detection method.

#### 3.4.1. Phytochemicals in different rice samples

**Fig 3:**
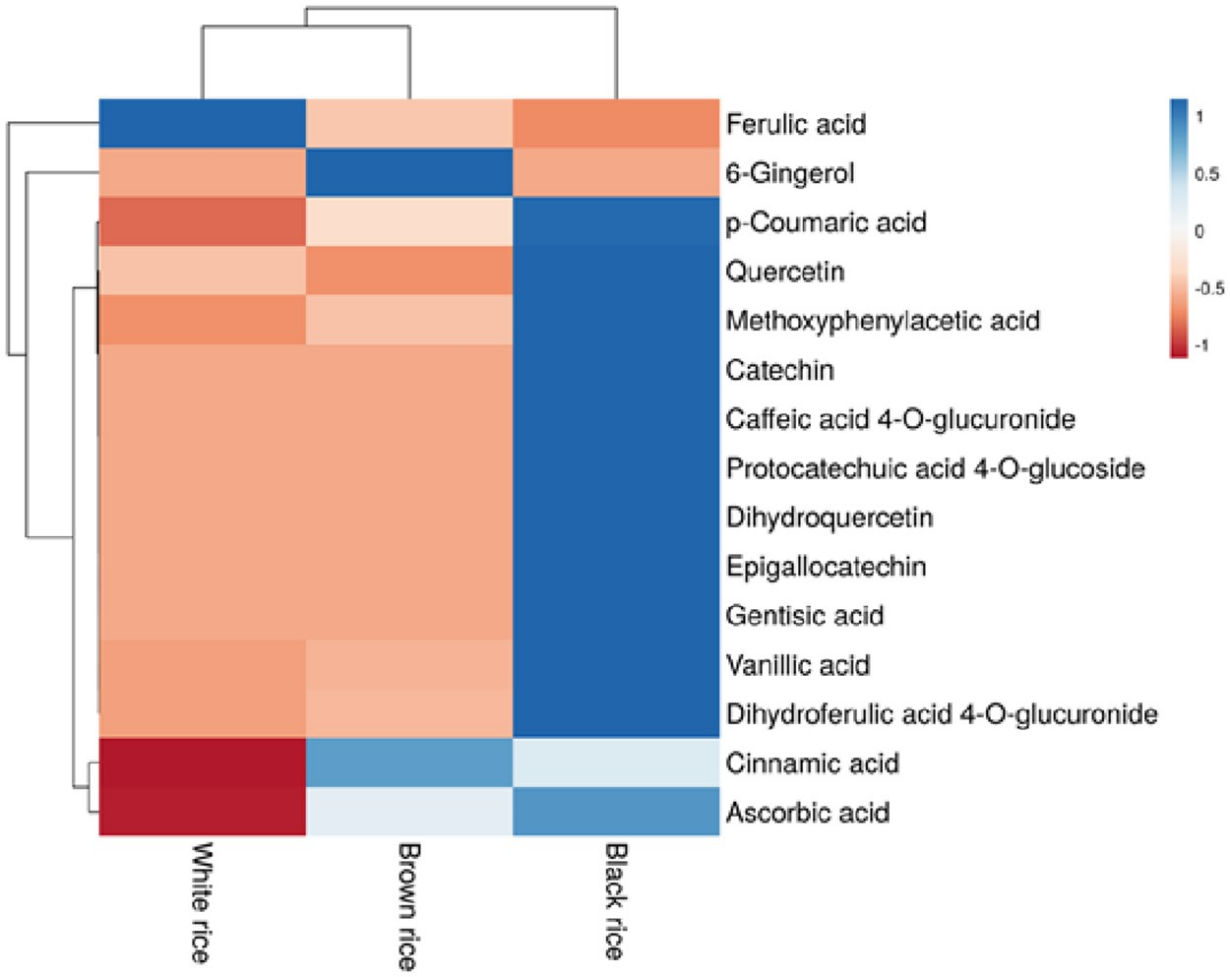
Heat map showing levels of phytochemicals in different pigmented and non-pigmented rice (white, brown, and black) extracts.

Dietary phenolics in a wide range of foods, beverages, fruit and cereals, known as phytotherapy chemicals, are recognized as bioactive components that are generally related to the protective activity for sustaining good health when ingested in a regular diet. Our current research identified fifteen phytochemicals in three distinct local rice varieties (black (chalheugmi), brown (hyunmi), and white (Baegilmi)) using UHPLC-Q-TOF-MS^2^. It has been claimed that phenolic chemicals are responsible for the health advantages of whole-grain rice intake in the prevention of chronic illnesses. Five authentic available standards: ascorbic acid, p-coumaric acid, catechin, ferulic acid and quercetin, were used to quantify their concentrations in three rice varieties (S3 Fig). These common standards were applied for quantification as in previous research; these compounds have shown their potential as strong antioxidants and oxidative stress-induced disease reduction [3–5]. The content of the phytochemical compounds is shown in Tables 2 & 3. Ascorbic acid (244.3 μg/mL), p-Coumaric acid (45.3 μg/mL) and quercetin (19.2 μg/mL) were found most abundant in black rice. Catechin was detected only in black rice (7 μg/mL). However, white rice was found abundant in ferulic acid (158.3 μg/mL), followed by brown and black rice. Previous research has shown that ferulic acid is the most abundant phenolic acid in rice cultivars [29]. The current study found that ferulic acid was not the most prevalent phenolic acid in all rice varieties, as it was found highest in white rice only. Instead, p-coumaric acid, ascorbic acid, and quercetin were the most predominant phenolic acids in black rice, followed by brown rice. The differences in results can be ascribed to the diverse rice types and extraction procedures utilized in each research. Later, by comparing our findings to mass spectral literature evidence, we tentatively or positively identified ten phenolic compounds from white, brown and black rice soluble extracts, as shown in Table 3. Phenolics were identified and cross-referenced to other accessible spectrum databases; Metlin and Metabolomics Workbench are two examples of such databases. Most of the tentative phenolic compounds like gentisic acid, caffeic acid, vanillic acid, dihydroquercetin and cinnamic acid were abundant in black rice. Heatmap analysis was utilized to group phenolic phytochemicals based on concentrations (Fig 3), with the blue-to-red colour scheme representing decreasing concentration. Results demonstrated that black rice was most abundant in phenolic phytochemicals, followed by brown and white rice.

**Table 2:** Quantification of phytochemicals discovered in the free fractions of white, brown and black rice by UHPLC-Q-TOF-MS^2^.

S. No	Sample Name	Retention Time (Min)	Adduct/Charge	Detected MS/MS Concentration (μg/mL)	Formula	Name
**1**	White rice	N/A	N/A	N/A	C_6_H_8_O_6_	Ascorbic acid
	Brown rice	1.05	[M+H]+	160.8		
	Black rice	1.06	[M+H]+	244.3		
**2**	White rice	16.08	[M−H]−	19.2	C_9_H_8_O_3_	p-Coumaric acid
	Brown rice	16.09	[M−H]−	26.7		
	Black rice	16.09	[M−H]−	45.3		
**3**	White rice	N/A	N/A	N/A	C_15_H_14_O_6_	Catechin
	Brown rice	N/A	N/A	N/A		
	Black rice	10.11	[M−H]−	7		
**4**	White rice	16.71	[M−H]−	158.3	C_10_H_10_O_4_	Ferulic acid
	Brown rice	16.73	[M−H]−	94		
	Black rice	16.72	[M−H]−	83.9		
**5**	White rice	19.34	[M+H]−	4.6	C_15_H_10_O_7_	Quercetin
	Brown rice	19.36	[M−H]−	1.9		
	Black rice	19.34	[M−H]−	19.2		

Nd-Not determined

**Table 3:** Tentative phenolic phytochemicals discovered in the free fractions of white, brown and black rice by UHPLC-Q-TOF-MS^2^.

S.No	Sample Name	RT (min)	Peak Area	Charge /Adduct	Precursor mass	Found at mass	Molecular Formula	Tentative Phenolic compound
**1**	WR(white rice)	Nd	Nd	[M‒H]‒	Nd	Nd	C_15_H_14_O_7_	Epigallocatechin
	BR(Brown rice)	Nd	Nd	[M‒H]‒	Nd	Nd		
	BLK(Black rice)	5.49	780000	[M‒H]‒	305.041	305.038		
**2**	WR(white rice)	Nd	Nd	[M+H]+	Nd	Nd	C_7_H_6_O_4_	Gentisic acid
	BR(Brown rice)	Nd	Nd	[M+H]+	Nd	Nd		
	BLK(Black rice)	5.49	1000000	[M+H]+	153.019	153.019		
**3**	WR(white rice)	Nd	Nd	[M‒H]‒	Nd	Nd	C_17_H_26_O_4_	6-Gingerol
	BR(Brown rice)	4.61	12000	[M‒H]‒	293.177	293.176		
	BLK(Black rice)	Nd	Nd	[M‒H]‒	Nd	Nd		
**4**	WR(white rice)	Nd	Nd	[M+H]+	Nd	Nd	C_8_H_8_O_4_	Vanillic acid
	BR(Brown rice)	11.71	7000	[M+H]+	167.032	167.033		
	BLK(Black rice)	11.73	120000	[M+H]+	167.032	167.035		
**5**	WR(white rice)	Nd	Nd	[M+H]+	Nd	Nd	C_15_H_12_O_7_	Dihydroquercetin
	BR(Brown rice)	Nd	Nd	[M+H]+	Nd	Nd		
	BLK(Black rice)	16.83	380000	[M+H]+	303.049	303.051		
**6**	WR(white rice)	Nd	Nd	[M+H]+	Nd	Nd	C_13_H_16_O_9_	Protocatechuic acid 4-O-glucoside
	BR(Brown rice)	Nd	Nd	[M+H]+	Nd	Nd		
	BLK(Black rice)	1.24	140000	[M+H]+	315.071	315.072		
**7**	WR(white rice)	Nd	Nd	[M+H]+	Nd	Nd	C_15_H_18_O_9_	Caffeic acid 4-O-glucuronide
	BR(Brown rice)	Nd	Nd	[M+H]+	Nd	Nd		
	BLK(Black rice)	8.54	98000	[M+H]+	355.067	355.067		
**8**	WR(white rice)	Nd	Nd	[M‒H]‒	Nd	Nd	C_16_H_20_O_10_	Dihydroferulic acid 4-O-glucuronide
	BR(Brown rice)	14.80	8600	[M‒H]‒	371.097	371.097		
	BLK(Black rice)	14.81	140000	[M‒H]‒	371.097	371.098		
**9**	WR(white rice)	Nd	Nd	[M‒H]‒	Nd	Nd	C_9_H_8_O_2_	Cinnamic acid
	BR(Brown rice)	4.03	7800	[M‒H]‒	147.046	147.0455		
	BLK(Black rice)	4.01	32000	[M‒H]‒	147.046	147.0456		
**10**	WR(white rice)	Nd	Nd	[M‒H]‒	Nd	Nd	C_9_H_10_O_3_	Methoxyphenylacetic acid
	BR(Brown rice)	15.28	5800	[M‒H]‒	165.057	165.0558		
	BLK(Black rice)	15.29	49000	[M‒H]‒	165.057	165.056		

Nd-Not determined, RT-Retention time.

Our study shows that pigmented rice varieties have much greater phenolic content than non-pigmented rice, which correlates with better antioxidant activity and makes black rice a potential sample for the functional food industry. As a consequence, the findings of this study accord with earlier findings showing that phenolic compounds in grains were predominantly responsible for rice grain antioxidant and other biological activities [17, 30].

The KEGG databases were used to demonstrate the early metabolic route of some of the phenolic compounds identified in our study (https://www.genome.jp/kegg/pathway.html) (S4 Fig). The phenylpropanoid biosynthesis pathway, the flavonoid biosynthetic pathway, and the flavones and flavonols biosynthetic pathway are the few pathways that govern the production of some of these compounds. Ten phenol metabolites, including cinnamic acid, p-coumaric acid, caffeic acid, ferulic acid, 5-hydroxyferulic acid, sikimic acid, and quinic acid, were mostly generated in the phenylpropanoid biosynthesis pathway. Meanwhile, four metabolites were mostly generated in the flavonoid metabolic pathway: epicatechin, (+)-catechin, epigallocatechin, and gallocatechin. The third flavone and flavonol biosynthesis pathway produced quercetin and its derivatives. As a result, we found that changes in the varieties and amounts of phenolic compounds in non-pigmented and pigmented rice may be linked to variances in the transition of distinct genes to crucial sites in the metabolic pathway. In later analysis, the metabolic pathways may be used to verify the transformation genes of certain particular phytochemicals triggering genes and supply suggestions for the selection and acquisition of outstanding variations.

#### 3.4.2. Principal component analysis (PCA) and correlation

S2 Table displays the results of Pearson’s correlation coefficient, which was used to assess the probable association between TPC, TFC, TAC, DPPH, ABTS, and FRAP radical scavenging activities. TPC exhibited a substantial positive correlation with TFC (r = 0.99918) and TAC (r = 0.99753) in our study. TPC, on the other hand, had a substantial negative correlation with DPPH (r = -0.78738), ABTS (r = -0.71389), and FRAP (r = -0.729) radical assays. Similarly, a substantial negative connection was seen between TFC and radical scavenging of DPPH (r = -0.76177), ABTS (r = -0.68494), and FRAP (r = -0.70068) radicals. In addition to TAC, there was a substantial negative connection with DPPH (r = -0.74215), ABTS (r = -0.66295), and FRAP (r = -0.67913) radicals. Nonetheless, DPPH radical scavenging activity had excellent linear correlations with ABTS and FRAP radical scavenging of 0.99379 and 0.99598, respectively. We have also included S5 Fig to better understand the correlations. In S5 Fig, blue depicts strong positive correlations with values closer to +1; red represents strong negative correlations with values closer to -1. Our findings support existing literature evidence of the antioxidant and phenolic correlating effects of polyphenols in cereal grains.

**Fig 4:**
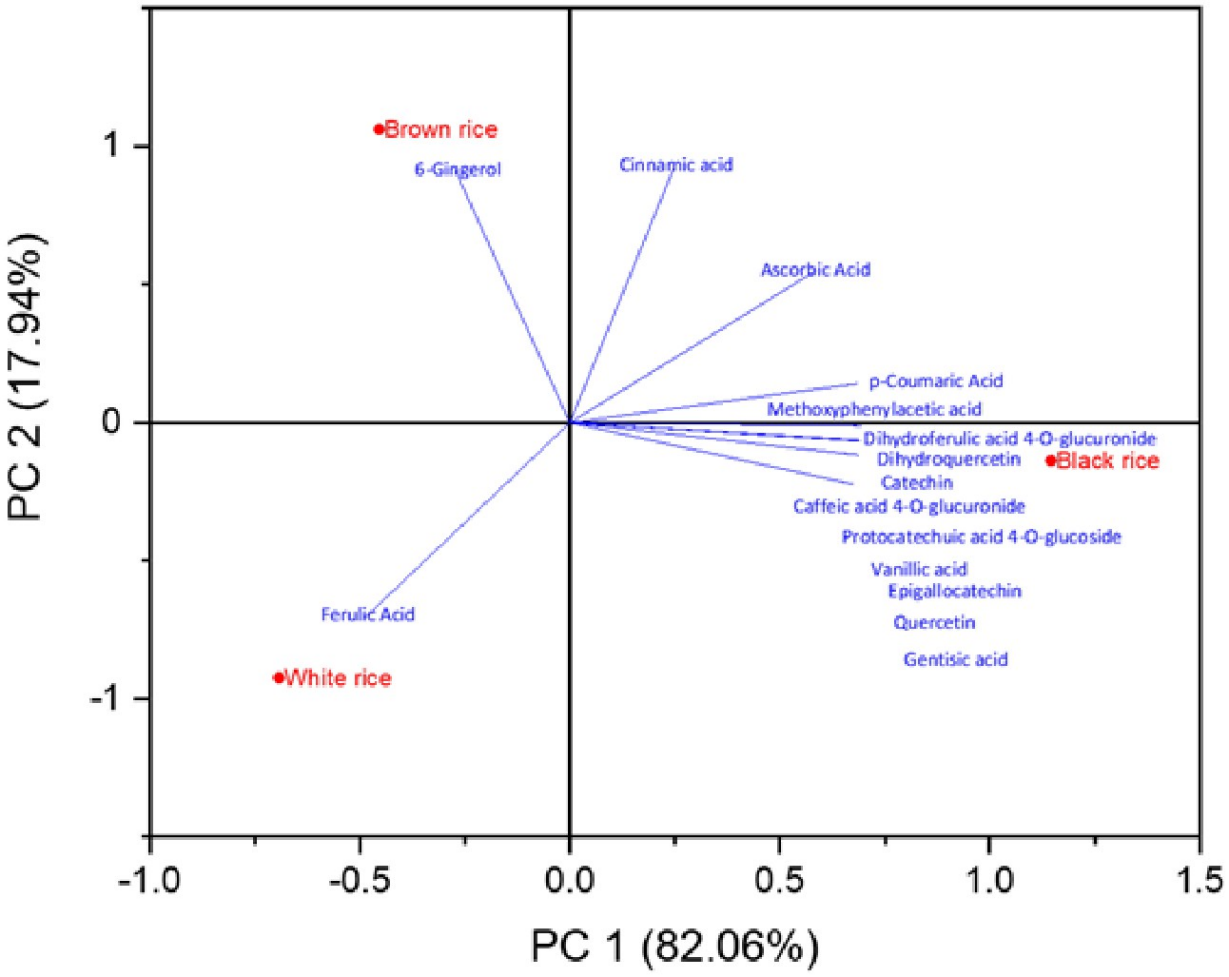
Principal component analysis (PCA) of rice samples (white, brown & black) was shown by comparing PC 1(component 1) with PC2 (component 2).

Principal component analysis (PCA) has also been used to comprehend better and prevent multicollinearity (Fig 4) and determine the distinctive feature of three rice types. PCA showed that all rice samples were divergent from each other as the majority of phenolic phytochemicals were identified in black rice, so more abundance of phenolics could be seen towards black rice; therefore, no correlation was found in PCA between the white, brown and black rice samples.

## 4. Conclusion

The *in-vitro* antioxidant activity, *ex-vivo* cellular antioxidant activity and identification of phytochemicals using UHPLC-Q-TOF-MS^2^ of three tested rice varieties, namely, black (chalheugmi), brown (hyunmi), and white (Baegilmi) rice were described in this study. The antioxidant activities (DPPH, ABTS and FRAP), CAAs, and concentration of TPC, TFC and TAC components changed substantially between tested samples. Tested black rice sample and ascorbic acid showed similar cellular antioxidant ability in CAAs. Also, a total of 15 phytochemical compounds were identified among three rice varieties. The highest quantities of phenolic phytochemicals were found in black rice samples that can deliver therapeutic or preventative benefits over various non-infectious chronic illnesses, including cardiovascular disease, cancer, obesity, and type II diabetes [31]. The findings of this work indicated the potential of black rice for enhancing human health through increased consumption and its application in the development of food products. However, more research is required to compare the tested local varieties with other universal rice varieties, which is planned in our future work. Additionally, *in-vivo* research on the capacity of a tested local black rice variety to reduce oxidative stress-related illnesses is also necessary for further validation of their health-promoting properties and functional food development.

## Supporting information

S1 Fig Different local Korean rice varieties. (TIF)

S2 Fig Effect of various rice (white, brown & black) extracts on the viability of Caco-2 cells was studied using the Ez cytox test kit. For 12 h, cells were exposed to an increasing concentration of rice extracts. Data are shown as means ± standard deviations (n = 3).
(TIF)

S3 Fig Phytochemical compounds identified in the ethanol extracts of white, brown and black rice by UHPLC-Q-TOF-MS^2^. (TIF)

S4 Fig The preliminary metabolic or biosynthesis pathway of some of the phenolic compounds identified in pigmented and non-pigmented rice varieties. (TIF)

S5 Fig Pearson correlation coefficients of total phenolic content, flavonoid content, anthocyanin and antioxidant capacities in different rice varieties ethanol extracts. (TIF)

S1 Table Table showing name, characteristics, area and color information of three tested local Korean rice varieties. (TIF)

S2 Table Pearson correlation coefficients of total phenolic content, flavonoid content, anthocyanin and antioxidant capacities in different rice varieties ethanol extracts. (TIF)
