# Supplementary figures and images for: Phytochemical profiling and cellular antioxidant efficacy of different rice varieties in colorectal adenocarcinoma cells exposed to oxidative stress
Source: PLoS One. 2022 Jun 3;17(6):e0269403. doi: 10.1371/journal.pone.0269403 (PMC9165792; doi:10.1371/journal.pone.0269403)

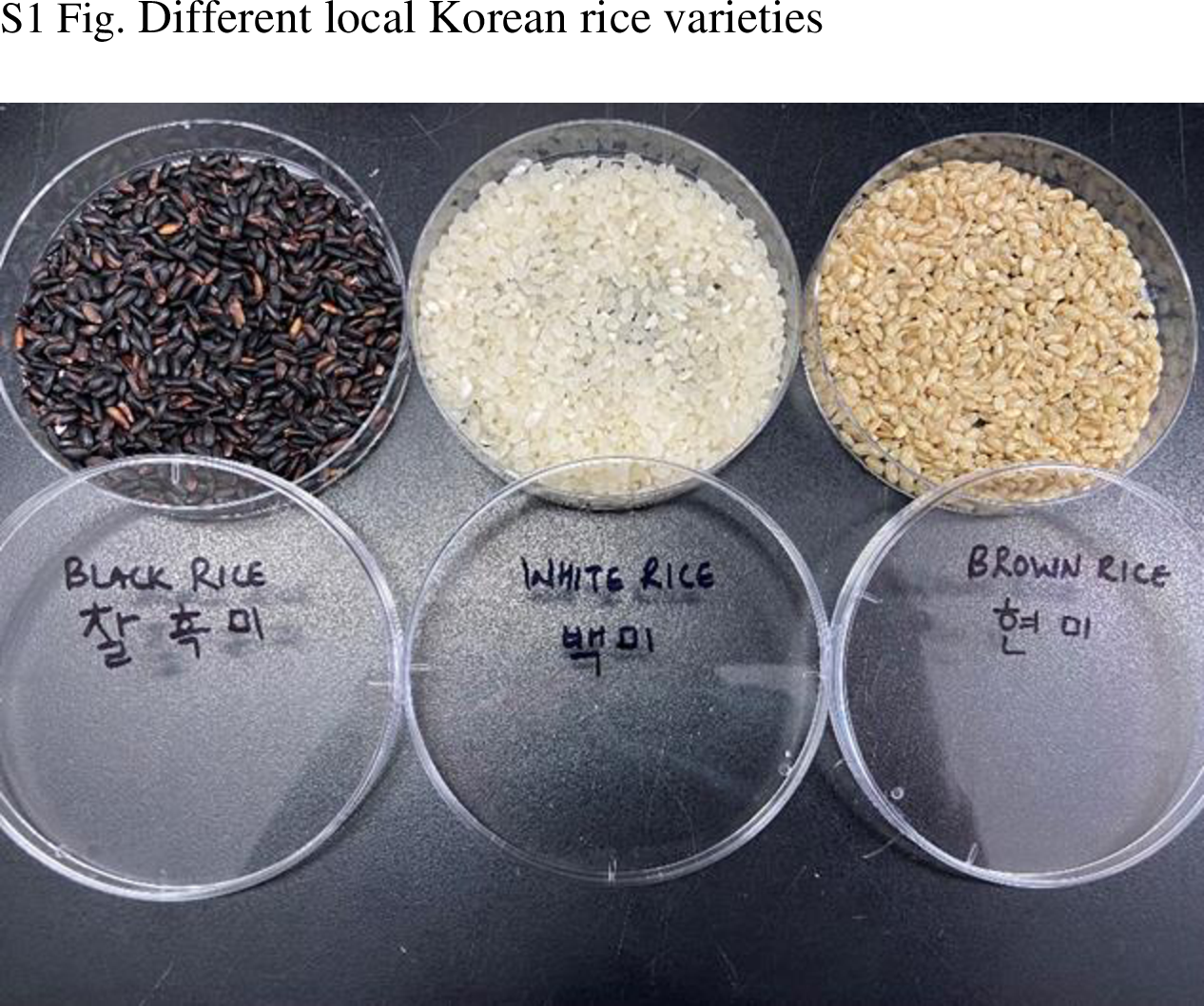

Supplement: S1 Fig — (TIF) [file pone.0269403.s001.tif]

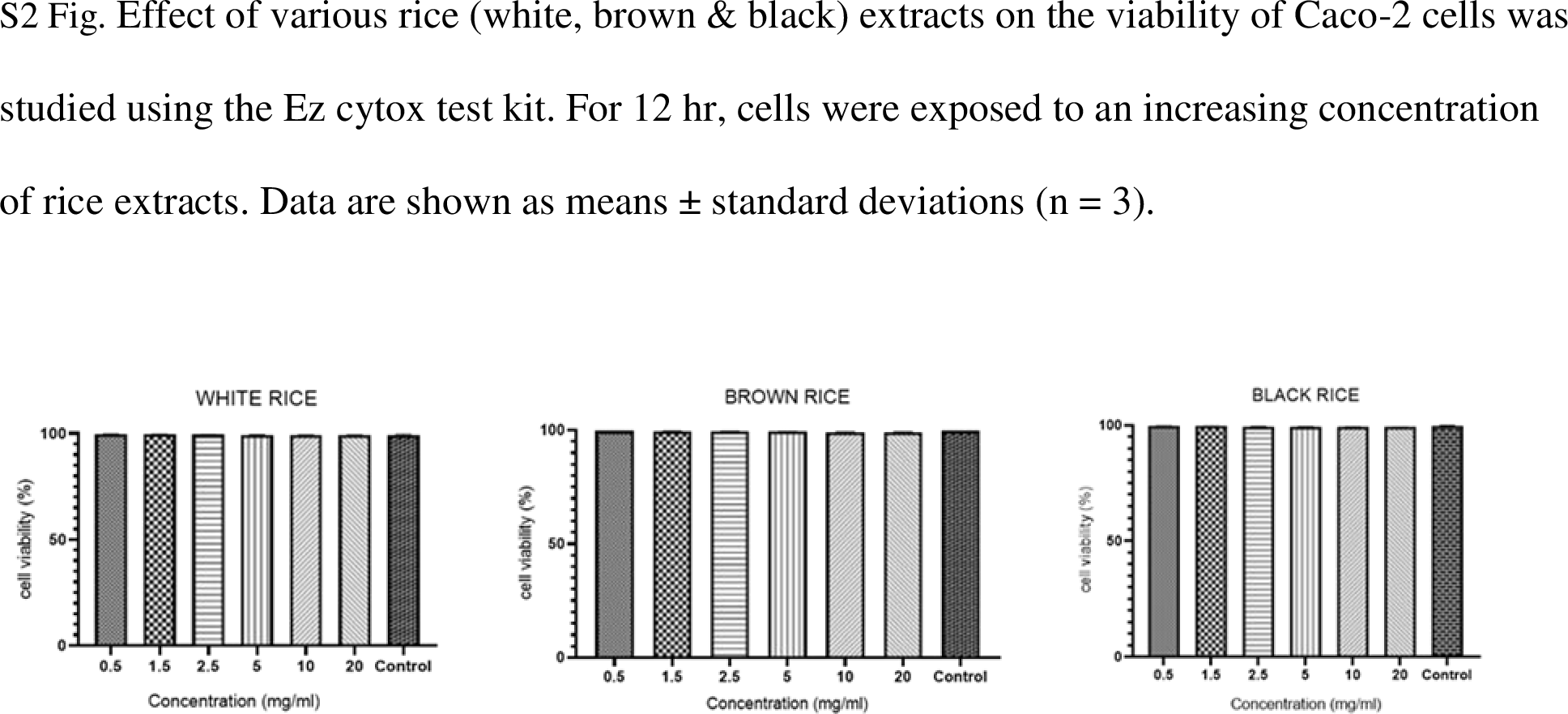

Supplement: S2 Fig — Data are shown as means ± standard deviations (n = 3). (TIF) [file pone.0269403.s002.tif]

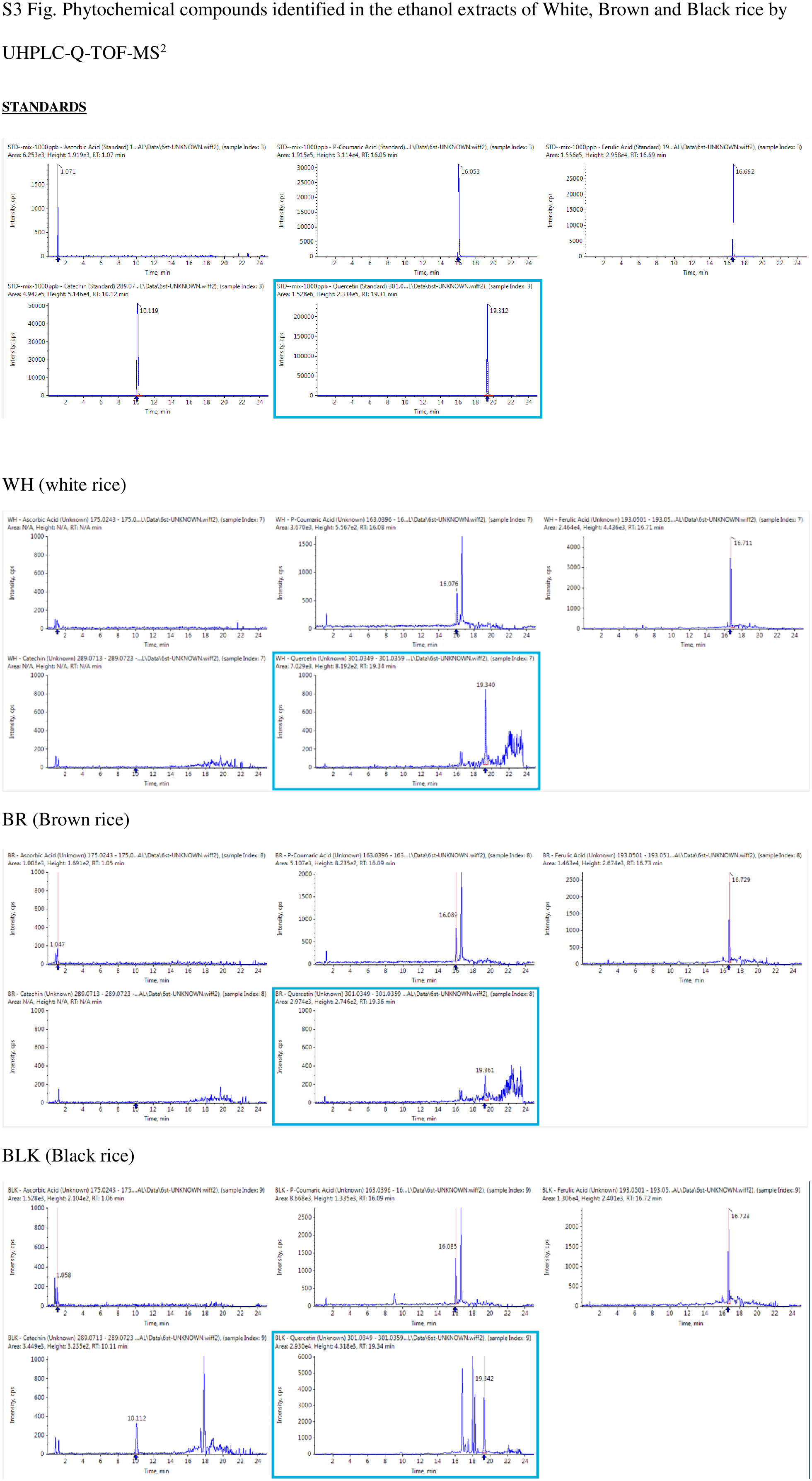

Supplement: S3 Fig — (TIF) [file pone.0269403.s003.tif]

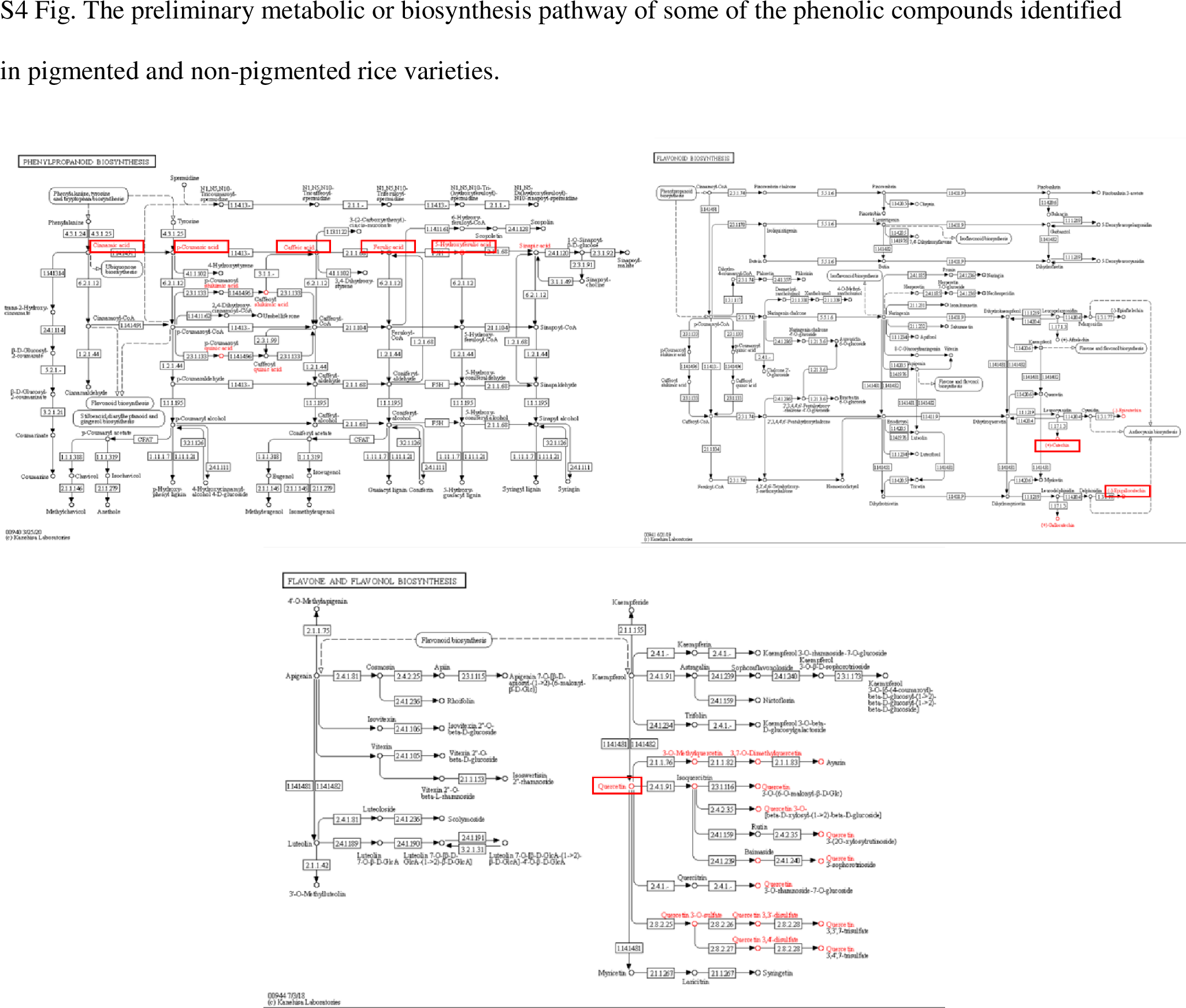

Supplement: S4 Fig — (TIF) [file pone.0269403.s004.tif]

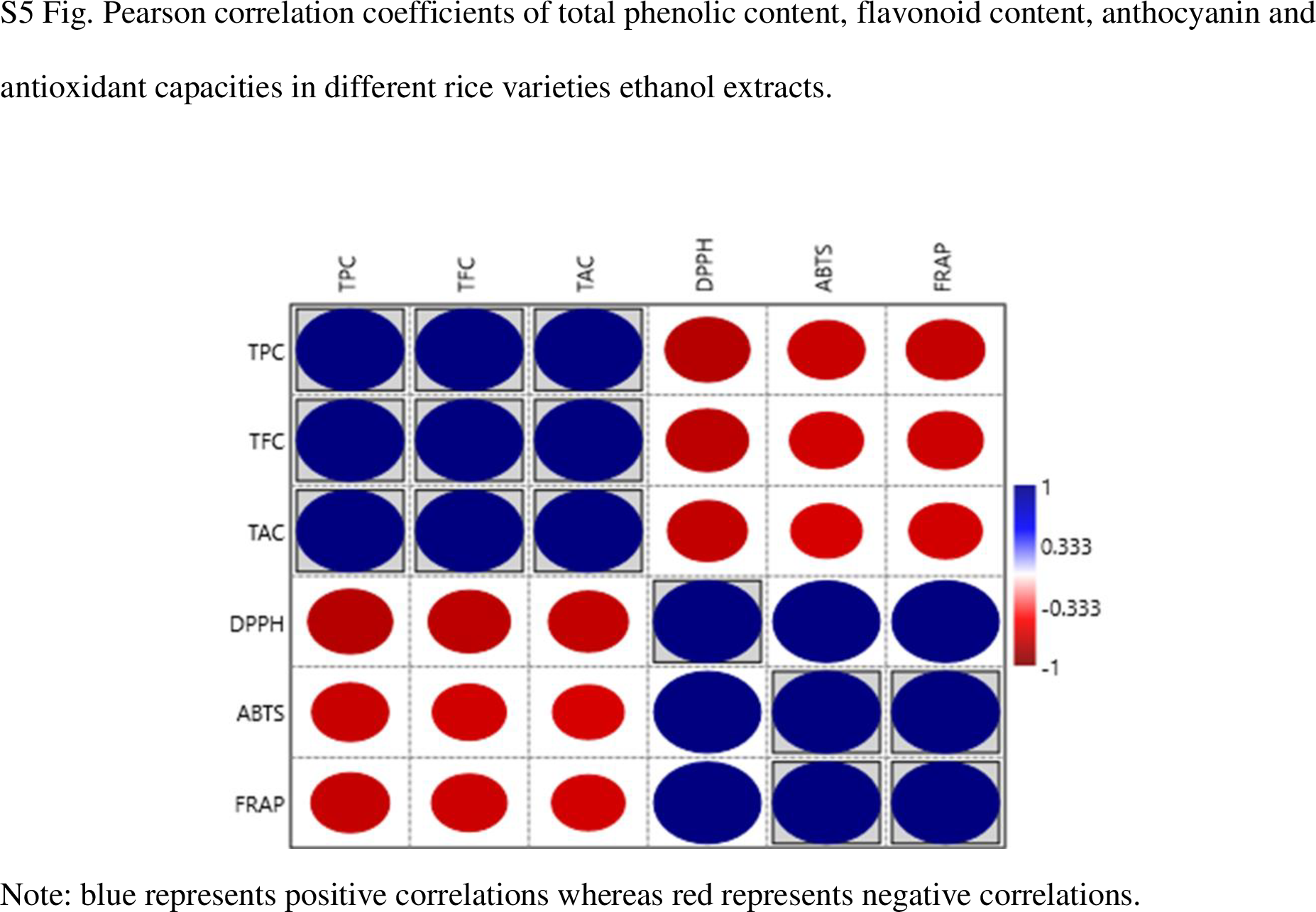

Supplement: S5 Fig — (TIF) [file pone.0269403.s005.tif]

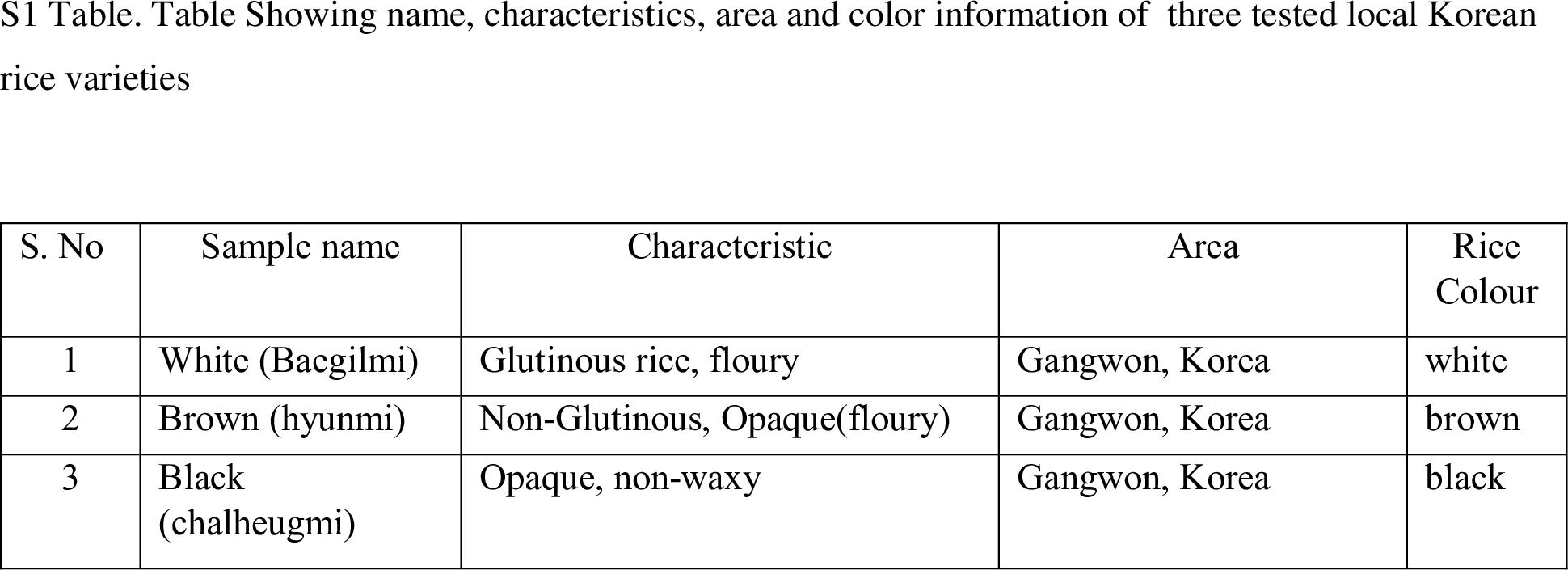

Supplement: S1 Table — (TIF) [file pone.0269403.s006.tif]

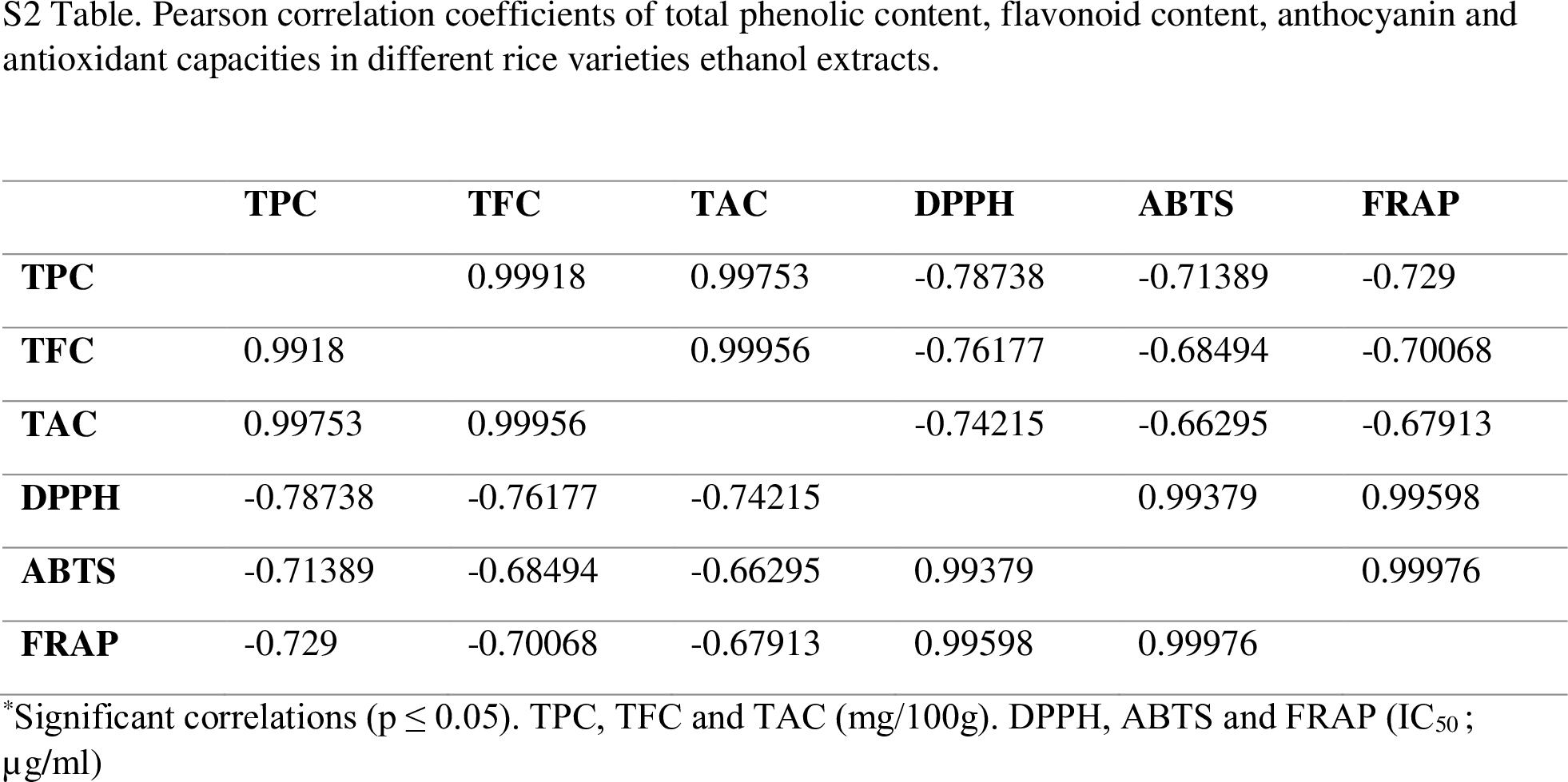

Supplement: S2 Table — (TIF) [file pone.0269403.s007.tif]
